# A Na^+^ Superionic Conductor for Room-Temperature Sodium Batteries

**DOI:** 10.1038/srep32330

**Published:** 2016-08-30

**Authors:** Shufeng Song, Hai M. Duong, Alexander M. Korsunsky, Ning Hu, Li Lu

**Affiliations:** 1 Materials Science Group, Department of Mechanical Engineering, National University of Singapore, 117575 Singapore; 2 College of Aerospace Engineering, Chongqing University, Chongqing 400044, P.R. China; 3 Multi-Beam Laboratory for Engineering Microscopy (MBLEM), Department of Engineering Science, University of Oxford, Parks Road, Oxford OX1 3PJ, United Kingdom

## Abstract

Rechargeable lithium ion batteries have ruled the consumer electronics market for the past 20 years and have great significance in the growing number of electric vehicles and stationary energy storage applications. However, in addition to concerns about electrochemical performance, the limited availability of lithium is gradually becoming an important issue for further continued use and development of lithium ion batteries. Therefore, a significant shift in attention has been taking place towards new types of rechargeable batteries such as sodium-based systems that have low cost. Another important aspect of sodium battery is its potential compatibility with the all-solid-state design where solid electrolyte is used to replace liquid one, leading to simple battery design, long life span, and excellent safety. The key to the success of all-solid-state battery design is the challenge of finding solid electrolytes possessing acceptable high ionic conductivities at room temperature. Herein, we report a novel sodium superionic conductor with NASICON structure, Na_3.1_Zr_1.95_Mg_0.05_Si_2_PO_12_ that shows high room-temperature ionic conductivity of 3.5 × 10^−3^ S cm^−1^. We also report successful fabrication of a room-temperature solid-state Na-S cell using this conductor.

Since their introduction in 1991, rechargeable lithium-ion batteries have proliferated throughout consumer electronics. They continue doing so apace, alongside increasing use in stationary energy storage applications. In considering future rechargeable batteries, the increasing lithium consumption and the likely future costs must be taken into account. Sodium (Na) has redox potential of *E*_*o*_ = −2.71 V *vs.* standard hydrogen electrode, which is close to that of Li. Na-based batteries have recently began making a comeback as an alternative for large-scale energy storage^1^,^2^,^3^.

In the development of oxide-based batteries, a principal long-standing challenge is associated with the ionic conductivities of solid electrolytes, poor mixed ionic-electronic conductivities in the electrodes, and inferior interfacial contact between electrolytes and electrodes^4^. This explains the reason why, despite increasing interest in solid-state electrolytes, little progress in solid-state batteries has been reported, with only few publications on oxide-based solid-state sodium batteries^5^,^6^,^7^. In the present work, the fabrication of a room temperature solid-state Na-S battery using an oxide electrolyte is demonstrated that represents the first step towards the creation of a solid-state room-temperature Na-S battery.

The development of sodium batteries began with 1960s, when a sodium beta-Al_2_O_3_ (NaAl_11_O_17_) solid electrolyte was first reported^8^, which inspired intense interest in the solid-state electrochemistry of Na-conducting solid electrolytes. However, the high operation temperature (300 to 350 °C) required for this system has raised a series of concerns over battery design and manufacture, safety issues as well as maintenance costs. For example, the extremely corrosive polysulphide melts, and the degradation of sodium beta-Al_2_O_3_ at the high operation temperature, can potentially result in battery failure, leading to fire hazard, and in some cases causing explosion^9^,^10^. Decreasing the battery working temperature would enhance battery safety, improve durability, and reduce cost^11^. It is particularly desirable for a solid-state Na-based battery to operate at ambient temperature. Unfortunately, to date no superior solid-state electrolytes have been reported for room-temperature Na-based battery operation, in particular for the Na-S system. A promising sulphide glass-ceramic electrolyte with a conductivity of 10^−4^ S cm^−1^ at room temperature, has recently been reported by Tatsumisago’s group^12^. However, a practically useful value of 10^−3^ S cm^−1^ is required to enable realistic battery design^13^. Four decades ago, pioneering work by Hong and Goodenough on NASICON (sodium super ion conductor) structure, Na_1+x_Zr_2_Si_x_P_3−x_O_12_ (0 ≤ x ≤ 3), demonstrated that good room-temperature conductivities (~10^−4^ S cm^−1^) could be achieved, owing to the presence of a 3D cation transportation channel^14^,^15^. Surprisingly, this remarkable opening for further research in the field appears to have been ignored so far.

Previously, we studied Li-ion electrolytes with garnet structure^16^. The alkaline earth cations have long been viewed as confined to being located exclusively at the dodecahedral 8-coordination sites (La sites) in the garnet structure owing to their large ionic radii^17^,^18^. However, we found that alkaline earth metals can be made to occupy the octahedral 6-coordination sites (Zr sites) through a structural transformation by a facile mechanochemical method, leading to enhanced room-temperature conductivity^19^. We also note a report of conductivity enhancement in NASICON electrolytes to which metal oxides (for example, Y_2_O_3_, TiO_2_, SnO_2_, V_2_O_5_, Nb_2_O_5_, Ta_2_O_5_, MgO, ZnO) have been added, although the room-temperature conductivities were not studied in these systems^20^. Mindful of previous research reports, we describe here the development and characterization of NASICON electrolytes doped with alkaline earth ions at octahedral 6-coordination Zr sites through mechanochemical synthesis. Specifically, the prepared electrolyte with a composition of Na_3.1_Zr_1.95_Mg_0.05_Si_2_PO_12_ presents a high room-temperature conductivity at the level of 10^−3^ S cm^−1^.

## Results

### Materials synthesis

**Figure 1:**
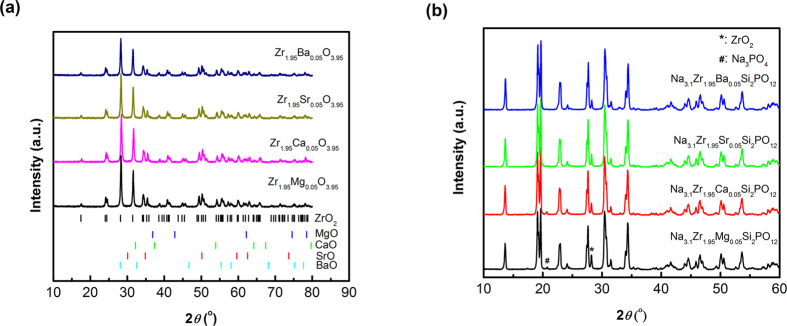
XRD patterns of (**a**) solid solutions of ZrO_2_ and MO (M = Mg, Ca, Sr, Ba) after high energy ball milling for 2 h. (**b**) Na_3.1_Zr_1.95_M_0.05_Si_2_PO_12_ (M = Mg, Ca, Sr, Ba) sintered at 1260 °C for 16 h.

Na_3.1_Zr_1.95_M_0.05_Si_2_PO_12_ (M = Mg, Ca, Sr, Ba) were synthesized by doping with alkaline earth ions at octahedral 6-coordination Zr sites. The procedure employed in this work consists of two sequential steps. Firstly, solid solutions of alkaline earth metal oxides (MO) and ZrO_2_ were synthesized by high energy ball milling at 875 rpm for 2 h (SPEX SamplePrep 8000 M Mixer). Then NASICON Na_3.1_Zr_1.95_M_0.05_Si_2_PO_12_ structures were synthesized through solid-state reaction of Na_2_CO_3_, Zr_1.95_M_0.05_O_3.95_, SiO_2_, and NH_4_H_2_PO_4_ at 1260 °C. X-ray diffraction patterns (Fig. 1a) show that the matrix of Zr_1.95_M_0.05_O_3.95_ is ZrO_2_, rather than MO. This provides a clear piece of evidence that mechanochemical synthesis leads to the formation of a solid solution of MO in ZrO_2_. Sintered Na_3.1_Zr_1.95_M_0.05_Si_2_PO_12_ gives rise to XRD patterns (Fig. 1b) that exhibit monoclinic C2/c NASICON phase structure with very low impurity level of ZrO_2_, ~3–5wt.% from Rietveld refinement of powder XRD data (Tables S1–S4).

### Materials characterization

**Figure 2:**
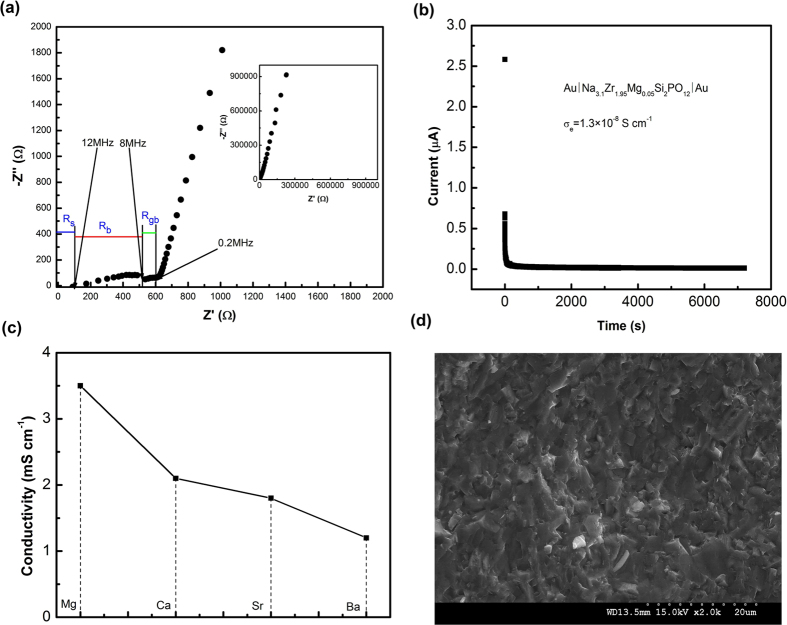
Characterization of Na_3.1_Zr_1.95_M_0.05_Si_2_PO_12_ (M = Mg, Ca, Sr, Ba) electrolytes. (**a**) Impedance plot of Na_3.1_Zr_1.95_Mg_0.05_Si_2_PO_12_ measured in Ar at room temperature. The inset is described from 12 MHz to 1 Hz. (**b**) Time dependence of dc current for Na_3.1_Zr_1.95_Mg_0.05_Si_2_PO_12_ pellet with applying a constant voltage of 1 V on the blocking electrodes. (**c**) Comparison of room-temperature conductivities of Na_3.1_Zr_1.95_M_0.05_Si_2_PO_12_. (**d**) SEM cross-section image of Na_3.1_Zr_1.95_Mg_0.05_Si_2_PO_12_.

Conductivities of sintered pellets of Na_3.1_Zr_1.95_Mg_0.05_Si_2_PO_12_ are evaluated by electrochemical impedance spectroscopy (EIS). Typical EIS plots as shown in Fig. 2a obtained using ion-blocking Au electrodes exhibit two semicircles and one tail in the high- and low-frequency range, suggesting that the investigated material is inherently ionically conductive and is bulk-boundary resistance separated. The EIS results resolve well the grain and grain-boundary resistances at room temperature in the frequency range of 12 MHz and 0.2 MHz. In Fig. 2a, one depressed semicircle in the higher frequency range (12–8 MHz) represents the bulk contribution, R_b_, which not start from “0” due to a contact resistance, R_s_ (~120 Ω), whereas, another semicircle in the lower frequency range (8–0.2 MHz) represents the grain-boundary contribution, R_gb_^21^. The total conductivity of the Na_3.1_Zr_1.95_Mg_0.05_Si_2_PO_12_ pellet (thickness of 2.7 mm and diameter of 4.5 mm) at room temperature is calculated to be ~3.5 × 10^−3^ S cm^−1^. An interesting observation is that the grain-boundary resistance is much lower than that of grain at room temperature. This demonstrates by far the best conductivity realized in a solid-state Na-ion electrolyte. Figure 2b shows the result of dc polarization measurement of sintered Na_3.1_Zr_1.95_Mg_0.05_Si_2_PO_12_ pellet with Au as blocking electrodes. The electronic conductivity ~1.3 × 10^−8^ S cm^−1^ at room temperature is detected by dc polarization technique. The sodium-ion transference number is close to one (t_Na+_ = (σ_total_ − σ_e_)/σ_total_ = 0.99999). The conductivities of Na_3.1_Zr_1.95_M_0.05_Si_2_PO_12_ (M = Ca, Sr, Ba) are lower than that of Na_3.1_Zr_1.95_Mg_0.05_Si_2_PO_12_ (Fig. 2c). The Na^+^-ion mobility and conductivity depend mainly on the crystal structure. The addition of alkaline earth ions modifies the crystal structure of NASICON phase because of their large ionic radii. Besides, the addition of alkaline earth metals promotes sintering, and induces well-crystallized grains and dense microstructure (Fig. 2d) helpful for ion conduction.

**Figure 3:**
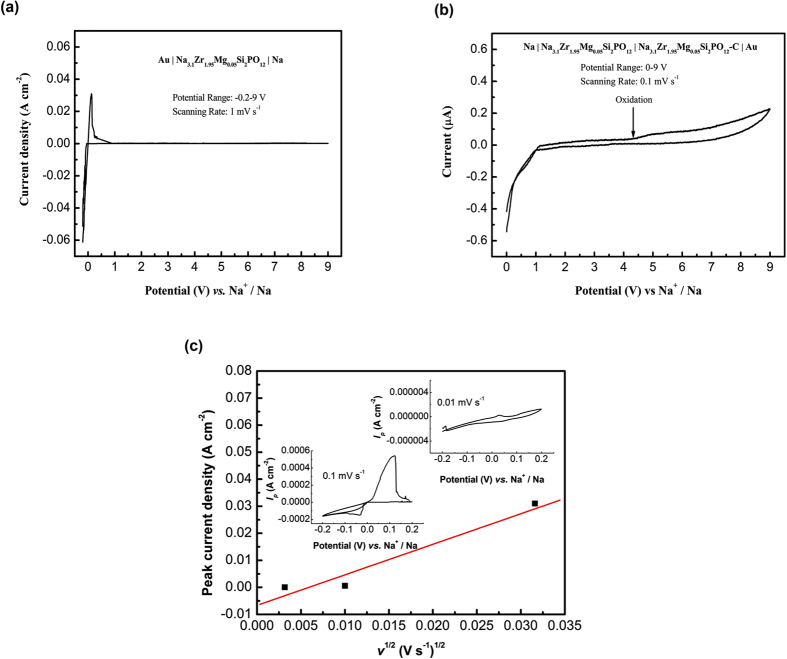
Cyclic voltammetry of Na_3.1_Zr_1.95_Mg_0.05_Si_2_PO_12_: (**a**) cyclic voltammetry of Au/Na_3.1_Zr_1.95_Mg_0.05_Si_2_PO_12_ pellet/Na cell from −0.2 to 9 V at scan rate of 1 mV s^−1^, (**b**) cyclic voltammetry of Au/Na_3.1_Zr_1.95_Mg_0.05_Si_2_PO_12_ pellet/Na_3.1_Zr_1.95_Mg_0.05_Si_2_PO_12_-carbon/Na cell from 0 to 9 V at scan rate of 0.1 mV s^−1^, and (**c**) Plot of peak current density *vs.* scan rate from the cyclic voltammetry. The inset is cyclic voltammetry curves at scan rate of 0.01 mV s^−1^ and 0.1 mV s^−1^.

Besides the high ionic conductivity, an essential requirement for solid electrolytes is a large electrochemical stability window. The electrochemical stability of the present material against metallic sodium is examined by cyclic voltammetry using a Au/Na_3.1_Zr_1.95_Mg_0.05_Si_2_PO_12_/Na cell with a scan range of −0.2 to 9 V and a scan rate of 1 mV s^−1^ (Fig. 3a). It is noted that Han *et al*.^22^ indicated that the wide electrochemical stability of lithium solid electrolytes is overestimated by the large lithium deposition/dissolution peaks via conventional experimental method with Li/electrolyte/inert metal semi-blocking electrode, and new experimental method is developed to evaluate the electrochemical stability of lithium solid electrolytes which uses Li/electrolyte/electrolyte-carbon/inert metal cell. In the present work, therefore Na/Na_3.1_Zr_1.95_Mg_0.05_Si_2_PO_12_/Na_3.1_Zr_1.95_Mg_0.05_Si_2_PO_12_-carbon/Au cell is also assembled to further evaluate the electrochemical stability window of Na_3.1_Zr_1.95_Mg_0.05_Si_2_PO_12_, with a scan range of 0 to 9 V (to avoid Na deposition/dissolution peaks) and a scan rate of 0.1 mV s^−1^. As shown in Fig. 3b, the oxidation of Na_3.1_Zr_1.95_Mg_0.05_Si_2_PO_12_ starts at about 4.5 V, but the current is very low with the maximum current of about 0.2 μA at scan voltage of 9 V, indicating that only a little bit of Na_3.1_Zr_1.95_Mg_0.05_Si_2_PO_12_ is oxidized. Therefore, the Na_3.1_Zr_1.95_Mg_0.05_Si_2_PO_12_ electrolyte possesses a practically useful electrochemical window of 0–4.5 V vs Na/Na^+^, which is sufficient to most of sodium cathodes.

The diffusion coefficient at room temperature can be calculated by Eq. 1 using the relation between the scan rate and peak current obtained from the slop of *i*_*p*_*/A vs. v*^1/2^ plot^23^,^24^.


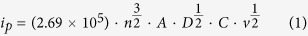


where *D* is the cation diffusion coefficient in cm^2^ s^−1^, *i*_*p*_ represents peak current in Amperes, *C* corresponds to the initial Na concentration in mol cm^−3^, *n* is the number of electrons per reaction species, *A* represents the electrode area in cm^2^, *v* represents the sweep rate in V s^−1^. Figure 3c shows the *i*_*p*_*/A vs. v*^1/2^ plot and it gives rise to the value of diffusion coefficient *D* of 5.24 × 10^−8^ cm^2^ s^−1^.

The ionic conductivity is calculated via the Stokes-Einstein relationship^25^,


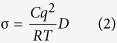


where *C*, *q*, *R*, and *T* are the Na concentration (0.019 mol cm^−3^), carrier charge, gas constant, and absolute temperature, respectively. The ionic conductivity is calculated to be 3.7 × 10^−3^ S cm^−1^, which is consistent with the conductivity value obtained from ac impedance method (3.5 × 10^−3^ S cm^−1^).

**Figure 4:**
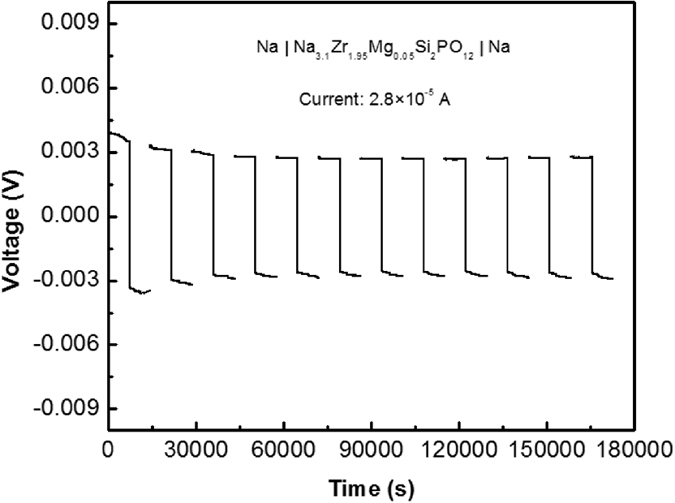
Galvanostatic cycling of symmetrical cells with sodium electrodes and Na_3.1_Zr_1.95_Mg_0.05_Si_2_PO_12_ electrolyte at the current density of 44 μA cm^−2^.

The Na/Na_3.1_Zr_1.95_Mg_0.05_Si_2_PO_12_/Na symmetric cells are galvanostatically cycled (Fig. 4). A minimal and stable polarization potential of 2.7 mV is obtained with current density of 44 μA cm^−2^. The direct-current conductivity of the present material is found to be 3.3 × 10^−3^ S cm^−1^ that is approximately consistent with the ac conductivity of 3.5 × 10^−3^ S cm^−1^ found by EIS, and the conductivity of 3.7 × 10^−3^ S cm^−1^ determined by cyclic voltammetry. Good agreement between different conductivity measurement methods (EIS, cyclic voltammetry, galvanostatic cycling), confirms the conclusion that the present material indeed possesses extremely high ionic conductivity in excess of 10^−3^ S cm^−1^ at room temperature.

**Figure 5:**
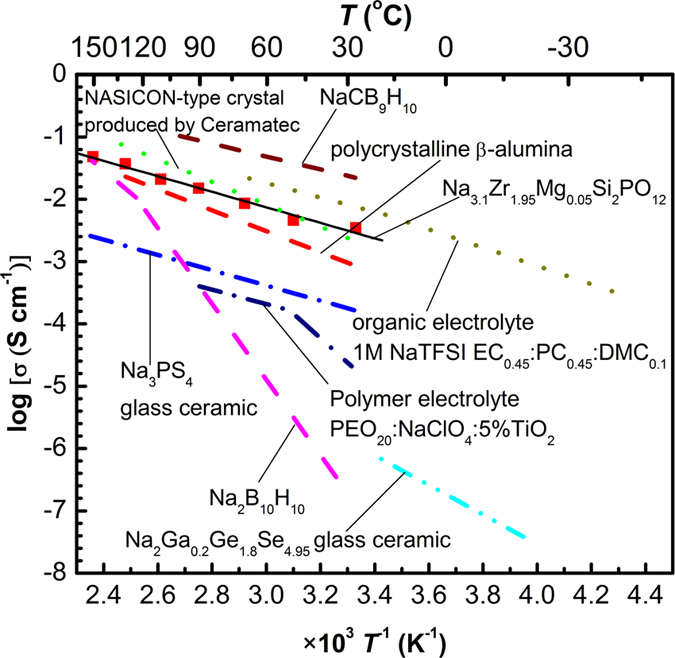
Temperature-dependent ionic conductivity of Na_3.1_Zr_1.95_Mg_0.05_Si_2_PO_12_ compared with other reported Na-ion conductors. ■: Na_3.1_Zr_1.95_Mg_0.05_Si_2_PO_12_ values. The solid line represents linearly fitted data.

Figure 5 shows the temperature-dependent total ionic conductivity of Na_3.1_Zr_1.95_Mg_0.05_Si_2_PO_12_ electrolyte together with those of other promising Na-ion electrolytes. The Na_3.1_Zr_1.95_Mg_0.05_Si_2_PO_12_ electrolyte is characterized by the conductivity of ~3.5 × 10^−3^ S cm^−1^ at room temperature and activation energy of ~0.25 eV. For example, the organic liquid electrolyte propylene carbonate–ethylene carbonate-dimethyl carbonate (45:45:10 wt.%) containing 1M NaTFSI (ref. 26) has conductivity around 10^−2^ S cm^−1^. Polymer electrolyte, such as PEO-NaClO_4_-TiO_2_ (ether-oxygen-to-sodium ratio was 20:1, addition of 5% TiO_2_) (ref. 27) has room-temperature conductivity of 10^−5^ S cm^−1^. Doped sodium hydride electrolyte, such as Na_2_B_10_H_10_ has conductivity of 10^−2^ S cm^−1^ only above 110 °C, whilst its conductivity drops to only ~5 × 10^−7^ S cm^−1^ at room temperature^28^. Exceptionally, NaCB_9_H_10_ is reported to have the highest conductivity of ~0.03 S/cm at room temperature^29^. The chalcogenide glass-ceramic electrolyte, such as Na_2_Ga_0.2_Ge_1.8_Se_4.95_ has conductivity barely exceeding 10^−5^ S cm^−1^ at room temperature^30^. The crystalline Na_3_PS_4_ glass ceramic has conductivity of 2.62 × 10^−4^ S cm^−1^ at room temperature^12^, which is the best reported for a sodium sulphide conductor. The classical β-alumina electrolyte and NASICON-type crystal produced by Ceramatec possesses room-temperature conductivity of ~10^−3^ S cm^−1^, but extremely high sintering temperatures of 1800 °C are needed for fabrication^31^,^32^. The present Na_3.1_Zr_1.95_Mg_0.05_Si_2_PO_12_ is characterized by practically useful conductivity of above 10^−3^ S cm^−1^.

### Application to all-solid-state sodium-sulphur batteries

**Figure 6:**
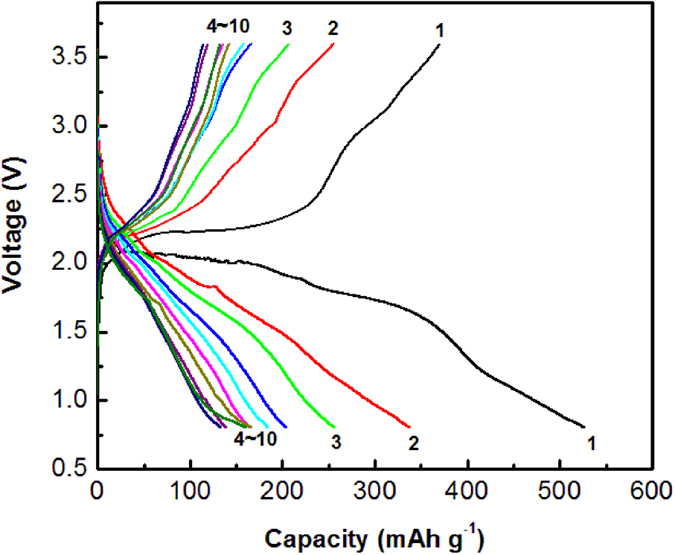
Charge-discharge profiles of a solid-state sodium battery (Na/Na_3.1_Zr_1.95_Mg_0.05_Si_2_PO_12_/S) at ambient temperature. The batteries were examined at a constant current of 8.92 μA (i.e., ~0.01C rate) with a cut-off voltage of 0.8–3.6 V.

Discovery of the new solid electrolyte with high conductivity helps to overcome the poor mixed ionic and electronic conductivities. To reduce interfacial impedance and segregation of Na^+^ ions along the interface, inorganic-organic hybrid cathode can be considered. To achieve this, the immobilized ionic liquid-based Na-ion polymer electrolyte is firstly synthesized by a mechanochemical reaction. Then, the sulphur-carbon composite is synthesized through mechanochemical milling sulphur and carbon at the weight ratio of 1:1. The sulphur-carbon composite is mechanochemically milled with polymer electrolyte at the weight ratio of 1:1. Solid-state sodium batteries are assembled in a coin cell consisting of a sulphur-carbon-polymer hybrid cathode, a Na_3.1_Zr_1.95_Mg_0.05_Si_2_PO_12_ electrolyte and a sodium anode. Figure 6 shows galvanostatic testing results of the Na-S battery (Na/Na_3.1_Zr_1.95_Mg_0.05_Si_2_PO_12_/S) operated with a cut-off voltage range of 0.8–3.6 V at the current of 8.92 μA (i.e., ~0.01C rate) at ambient temperature. The initial discharge capacity is about 527 mAh g^−1^. This capacity is higher than those of high-temperature Na-S cells using beta-alumina as electrolyte and ambient-temperature Na-S cells with traditional sulphur-carbon composite using organic liquid electrolyte^9^,^11^,^33^,^34^. The present cell experiences a sharp decrease in capacity during the initial 10 galvanostatic cycles, with the discharge capacity fading from 527 to 160 mAh g^−1^ during ten cycles. Severe capacity decrease is likely to be associated with the generation of reversible sodium polysulphides that resist oxidation during subsequent charging. This phenomenon is known to be a general problem in other sulphur-based batteries (i.e., high-temperature Na-S batteries and Li-S batteries)^2^. Specific directed effort is required to address this fading mechanism and to enhance the cell performance.

**Figure 7:**
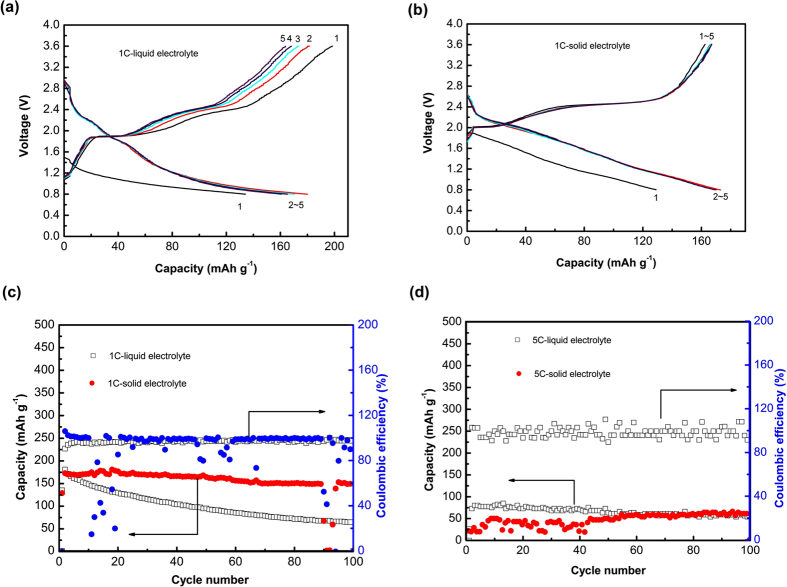
Electrochemical performance of two types of Na-S batteries using liquid electrolyte and solid-state electrolyte: (**a**) voltage profiles of cells using liquid electrolyte at 1C rate, (**b**) voltage profiles of cells using solid electrolyte at 1C rate, and (**c**) cycling performance of the two types of cells at (**c**) 1C rate and (**d**) 5C rate.

To evaluate the advantage of solid-state configuration to the liquid one, one cell is assembled with metallic sodium as anode, S-C composite as cathode, 1 M NaClO_4_/EC+PC (1:1 in weight) as liquid electrolyte, and glass microfiber as separator, whereas another cell using the present Na_3.1_Zr_1.95_Mg_0.05_Si_2_PO_12_ pellet (~1.0 mm) as electrolyte and separator is assembled. Figure 7 shows the electrochemical performance of the two types of Na-S batteries with a cut-off voltage range of 0.8–3.6 V at 1C and 5C rate. As shown in Fig. 7a,b, for the cells using liquid electrolyte and solid electrolyte respectively at 1C rate, there are no obvious discharge plateaus, but two charge plateaus at about 2.0 V and 2.4 V, which indicates a stepped oxidation reaction during charging process. The voltage profiles of solid-state cell coincide from 2^nd^ to 5^th^ cycle, indicating stable electrochemistry. Figure 7c reveals the highest initial capacity is ~180 mAh g^−1^, and the discharge capacity decays to ~64 mAh g^−1^ at 1C rate after 100 cycles with a capacity retention of only 36% of the cell using liquid electrolyte. For the solid-state one, the highest initial capacity is slight lower than that of liquid one, ~170 mAh g^−1^, but it delivers capacity of 150 mAh g^−1^ over 100 cycling with a capacity retention of 88%, indicating better cycling stability compared with the conventional Na-S batteries using liquid electrolyte. The Coulombic efficiency is maintained at ~100% except the first cycle for both cells. As shown in Fig. 7d, at 5C rate, the cell using liquid electrolyte delivers better initial capacity of ~84 mAh g^−1^, and fades mildly to ~54 mAh g^−1^ after 100 cycles. For the solid-state cell, the initial capacity is ~21 mAh g^−1^, and increase mildly to ~56 mAh g^−1^ after 60 cycles and ~60 mAh g^−1^ after 100 cycles, that is similar with the liquid one. Therefore, it is demonstrated that the Na-S cells using solid-state electrolyte can achieve better cycling stability than the liquid counterpart, because the solid-state electrolyte can efficiently depress the dissolution and shuttle of sodium polysufides in electrolyte.

## Discussion

**Figure 8:**
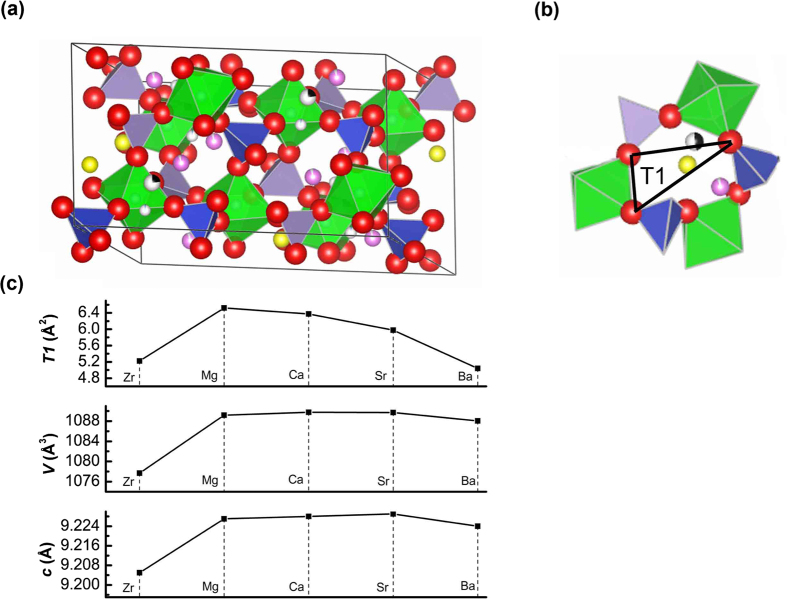
Refined crystal structure of Na_3.1_Zr_1.95_Mg_0.05_Si_2_PO_12_. (**a**) Polyhedral drawing of the unit cell of Na_3.1_Zr_1.95_Mg_0.05_Si_2_PO_12_. Na(1) ions: black spheres, Na(2) ions: yellow spheres, Na(3) ions: purple spheres. (**b**) A bottleneck of Na_3.1_Zr_1.95_Mg_0.05_Si_2_PO_12_ consisting of alternating three ZrO_6_ octahedra and three P/SiO_4_ tetrahedra. The triangle T1 is outlined. The Na(1), Na(2) and Na(3) ions are located below and above the bottleneck. (**c**) Lattice parameter, volume of unit cell and area of T1 of the Na_3_Zr_2_Si_2_PO_12_^14^,^36^ and Na_3.1_Zr_1.95_M_0.05_Si_2_PO_12_ (M = Mg, Ca, Sr, Ba).

Structural features play a key role in delivering fast ionic conduction. The crystal structure of the present material is examined via Rietveld refinement of powder XRD data (Fig. S1 and Tables S1–S4). The structure includes ZrO_6_ octahedra corner-sharing with P/SiO_4_ tetrahedra, with the alkaline earth ions located at Zr sites. One ZrO_6_ octahedron is combined with six P/SiO_4_ tetrahedra, forming a monoclinic framework with a three-dimensional Na^+^ ions channels (Fig. 8a). The Na^+^ ions occupy three types of sites, which are coordinated by O^2−^ ions as 6-fold Na(1) site, 8-fold Na(2) site and 5-fold Na(3) site, respectively^35^. Each Na(2) site connects two Na(1) sites, while each Na(1) site connects two Na(3) sites. There are no pathways among Na(2), Na(3) or Na(2) and Na(3) sites, owing to the long distances and/or polyhedra obstacles. We consider the possible efficient conduction pathway along the three-dimensional channels by selecting the shortest Na-Na hopping distances, that correspond to the lowest transport barrier^36^. For the Mg-doped system, the closest Na(1)-Na(2), Na(1)-Na(3), and Na(2)-Na(3) distances are 3.487 Å, 2.305 Å and 3.915 Å, respectively, which are more than twice the Na^+^ ionic radius (~1.02 Å). The Na(1)-Na(3) distance is the even shorter ~2.08 Å for Ca/Sr/Ba-doped systems, corresponding to approximately twice the Na^+^ ionic radius. This approch implies that the adjacent Na(1) and Na(3) sites may not be occupied simultaneously. The Na^+^ ions move toward the Na(1) vacancy, whilst, simultaneously the Na^+^ ions at the Na(3) sites migrate away, and vice versa. This suggests that the possible conduction pathway in monoclinic framework is the Na(2)-Na(1)-Na(3) pathway.

It can be surmised that the observed increase in the ionic conductivity associated with the ionic radii of octahedrally coordinated cations, the large ionic radii increase the lattice parameters and cell volume, thus facilitating the Na^+^ ion mobility^37^. In the present materials, the unit cell parameters and cell volume increase with the substitution of alkaline earth ions for Zr, but little variation is observed for different alkaline earth ions (Fig. 8c). In fact, the lattice parameters and ionic radii do not follow a monotonic relationship, reaching a maximum near Na content of 3–3.2 per formula unit^14^. To jump from one site to next one, Na^+^ ions must pass through a bottleneck as defined by Hong^14^. In the monoclinic NASICON framework, the bottleneck is a pseudo-hexagonal ring consisting of alternating three ZrO_6_ octahedra and three P/SiO_4_ tetrahedra (Fig. 8b). The bottleneck size is the principal factor controlling the activation energy and ionic conductivity. West *et al*.^38^ suggested a triangle T1 defined by the three O^2−^ ions to characterize the bottleneck size. We analyze the bottleneck in the pathway between the Na(2) and Na(1) sites using the area of triangle T1 (Fig. 8b). It can be seen that the area of triangle T1 increases with the substitution of alkaline earth ions for Zr, and decreases with the ionic radii of alkaline earth ions (Fig. 8c), indicating that the much larger alkaline earth ions narrow the bottleneck, thus decreasing the ionic conductivity. The Zr-Mg system has the largest area of triangle T1 (~6.522 Å^2^) that is significantly larger than that of the pure Zr system (~5.223 Å^2^), explaining the maximum ionic conductivity observed for the Zr-Mg system.

In summary, here we demonstrate that the Na_3.1_Zr_1.95_Mg_0.05_Si_2_PO_12_ with NASICON phase has superior room-temperature ionic conductivity of 3.5 × 10^−3^ S cm^−1^. We report ground-breaking progress in manufacturing a solid-state room temperature Na-S battery using Na_3.1_Zr_1.95_Mg_0.05_Si_2_PO_12_ solid electrolyte. Initial adequate cell performance is demonstrated, and further improvement can be sought by developing hybrid positive electrodes and by achieving good electrode-electrolyte contact. Moreover, we demonstrate that the present solid-state electrolyte can efficiently depress the dissolution and shuttle of sodium polysufides which leads to better cycling stability than the liquid counterpart. The present work will help to identify new strategies for developing organic-inorganic hybrid positive electrodes and chemically stable oxide-based electrolytes for the next generation of safe and inexpensive high-performance solid state sodium batteries.

## Methods

### Preparation of Na_3.1_Zr_1.95_M_0.05_Si_2_PO_12_ (M = Mg, Ca, Sr, Ba) electrolytes

The Na_3.1_Zr_1.95_M_0.05_Si_2_PO_12_ (M = Mg, Ca, Sr, Ba) were synthesized through solid-state reaction combined with mechanochemical synthesis. Firstly, The Zr_1.95_M_0.05_O_3.95_ solid solutions were prepared through mechanochemical reaction in a high energy ball mill for 2 h (SPEX SamplePrep 8000 M Mixer). The mixture of ZrO_2_ (Inframat Advanced Materials, ≥99.9%) and MO was milled by alternating 30 min of milling with 30 min in standby mode to avoid excessive heating. The solid electrolytes with the formula Na_3.1_Zr_1.95_M_0.05_Si_2_PO_12_ (M = Mg, Ca, Sr, Ba) were synthesized through solid-state reaction by mixing stoichiometric amounts of Na_2_CO_3_ (Sigma-Aldrich, ≥99.5%), SiO_2_ (Sigma-Aldrich, ≥99%), NH_4_H_2_PO_4_ (Sigma-Aldrich, ≥98%), and Zr_1.95_M_0.05_O_3.95_, and ball-milling with zirconium oxide balls for 2 h. The precursors were decomposed at 900 °C for 12 h in alumina crucibles, with repeated ball-milling for 2 h. The calcined powders were then cold pressed and sintered at 1260 °C for 16 h covered with the raw powders to avoid sodium loss.

### Characterization of solid electrolytes

The crystal structure was analyzed by Rietveld refinement of powder XRD data (Shimadzu XRD-6000 Cu-Kα), using GSAS software. The microstructure was examined on polished surfaces of the sintered pellet using SEM (S-4300 Shimadzu). The ionic conductivities were fixed by impedance spectroscopy measurements that were performed with a Solartron 1260+1287 System, applying AC potential of 10 mV from 32 MHz to 1 Hz in Ar atmosphere. Ion-blocking electrodes were formed by Au sputtering on both surfaces of the pellet. Measurement of DC conductivity was performed using sodium-electrolyte symmetric cell with constant current density of 44 μA cm^−2^ at room temperature inside an Ar-filled glove box. Sodium plates (~0.22 mm thickness) were attached to both faces of the pellet (2.0 mm thickness, 9.0 mm diameter), to serve as non-blocking electrodes. DC polarization was performed to evaluate the electronic conductivity and sodium-ion transference number. A Na_3.1_Zr_1.95_Mg_0.05_Si_2_PO_12_ pellet (3.5 mm thickness, 9.1 mm diameter) was sputtered by Au on both surfaces and a constant voltage of 1 V was applied. Cyclic voltammetry measurements were performed using two methods. The first method was using Au/Na_3.1_Zr_1.95_Mg_0.05_Si_2_PO_12_ pellet/Na semi-blocking cell to perform a linear sweep from −0.2 V to 9 V vs Na^+^/Na with varied scan rate of 1 mV s^−1^. The second method was using Na/Na_3.1_Zr_1.95_Mg_0.05_Si_2_PO_12_/Na_3.1_Zr_1.95_Mg_0.05_Si_2_PO_12_-carbon/Au cell to perform a linear sweep from 0 to 9 V vs Na^+^/Na with scan rate of 0.1 mV s^−1^ according to ref. 22. To make the Na/Na_3.1_Zr_1.95_Mg_0.05_Si_2_PO_12_/Na_3.1_Zr_1.95_Mg_0.05_Si_2_PO_12_-carbon/Au cell, the Na_3.1_Zr_1.95_Mg_0.05_Si_2_PO_12_-carbon electrode was prepared by mixing Na_3.1_Zr_1.95_Mg_0.05_Si_2_PO_12_ powder, carbon black, polyvinylidene fluoride with a weight ratio of 36:54:10, and n-methylpyrrolidinone to an electrode slurry. The slurry was then casted onto the polished Na_3.1_Zr_1.95_Mg_0.05_Si_2_PO_12_ pellet (~1.0 mm thickness and ~9 mm diameter) and dried at 120 °C overnight, then sputtered Au, after which, metallic sodium was attached on the other side of the pellet. The Na/Na_3.1_Zr_1.95_Mg_0.05_Si_2_PO_12_/Na_3.1_Zr_1.95_Mg_0.05_Si_2_PO_12_-carbon/Au cell was assembled using CR2025 coin cell and was performed cyclic voltammetry measurements on Solartron electrochemistry workstation.

### Characterization of solid-state Na-S batteries

A solid-state sodium battery was prepared with sulphur-carbon-polymer electrolyte hybrid material as cathode, Na_3.1_Zr_1.95_Mg_0.05_Si_2_PO_12_ ceramic pellet as electrolyte, sodium metal as anode. Firstly, the sulphur-carbon composite was prepared by mechanochemical milling with the sulphur to carbon weight ratio of 1:1 at 875 rpm for 5 h. The polymer electrolyte consisted of poly(ethylene oxide) (Sigma-Aldrich, 100 0000 g mol^−1^), NaClO_4_ (Sigma-Aldrich, ≥98%), SiO_2_ (Sigma-Aldrich, 5–15 nm particle size), 1-Ethyl-3-methylimidazolium bis(fluorosulfonyl)imide (Solvionic, H_2_O ≤ 20 ppm) was prepared by mechanochemical milling at 875 rpm for 1 h in a weight ratio of 0.53:0.074:0.06:1.33 in acetone. Following, the sulphur-carbon composite was added to the polymer electrolyte in a weight ratio of 1:1 and with mechanochemical milling at 875 rpm for 30 min. The slurry was then coated uniformly onto Na_3.1_Zr_1.95_Mg_0.05_Si_2_PO_12_ ceramic pellet (~1.2 mm thickness) and dried under vacuum at 50 °C overnight. Sodium foil was attached on the other surface of the Na_3.1_Zr_1.95_Mg_0.05_Si_2_PO_12_ ceramic pellet in Ar-filled glove box. Cell assembly was carried out in CR2025 coin cells. The charge-discharge measurements were conducted at a constant current of 8.92 μA (i.e., ~0.01C rate) with a cut-off voltage of 0.8–3.6 V on a MACCOR battery cycler at room temperature.

To evaluate the capability of present solid-state electrolyte compared with conventional liquid electrolyte, two different configuration of Na-S cells were constructed. The first type of Na-S cells used 1 M NaClO_4_ in ethylene carbonate (EC)/propylene carbonate (PC) (1:1 in weight) as electrolyte and Whatman GF/A fiber as separator, and the second type of Na-S cells used the present Na_3.1_Zr_1.95_Mg_0.05_Si_2_PO_12_ ceramic pellet (~1.0 mm thickness) as electrolyte and separator. The sulphur-carbon composite (1:1 in weight) was mixed with Super P conductive carbon (TIMCAL Ltd.) and polyvinylidene fuoride (PVDF, Sigma) at a weight ratio of 8:1:1 in N-methylpyrrolildone (NMP, Sigma) solvent to form uniform slurries and then was coated on Al foils to prepare the cathode. The cells were assmbled in Ar-filled glove box with metallic sodium as anode. To assemble cells using solid-state electrolyte, sodium was attached on a side of polished pellet and a very little drop of liquid electrolyte was used as a buffer between solid-state electrolyte and cathode. The charge-discharge measurements were conducted at a constant current of 1C and 5C rate with a cut-off voltage of 0.8–3.6 V.

## Additional Information

**How to cite this article**: Song, S. *et al*. A Na^+^ Superionic Conductor for Room-Temperature Sodium Batteries. *Sci. Rep.* **6**, 32330; doi: 10.1038/srep32330 (2016).

## Supplementary Material

Supplementary Information
