# Supplementary material for: A Na+ Superionic Conductor for Room-Temperature Sodium Batteries
Source: Sci Rep. 2016 Aug 30;6:32330. doi: 10.1038/srep32330 (PMC5004141; doi:10.1038/srep32330)
Supplement: Supplementary Information [file srep32330-s1.pdf]

# A $Na^+$ Superionic Conductor for Room-Temperature Sodium Batteries

**Shufeng Song[1,2], Hai M. Duong[1], Alexander M. Korsunsky[3], Ning Hu[2], Li Lu[1*]**

[1] Materials Science Group, Department of Mechanical Engineering National University of Singapore, Singapore 117575.

[2] College of Aerospace Engineering, Chongqing University, Chongqing 400044, P.R. China.

[3] Multi-Beam Laboratory for Engineering Microscopy (MBLEM), Department of Engineering Science, University of Oxford, Parks Road, Oxford OX1 3PJ, United Kingdom.

Correspondence and requests for materials should be addressed to L.L. (email: luli@nus.edu.sg).

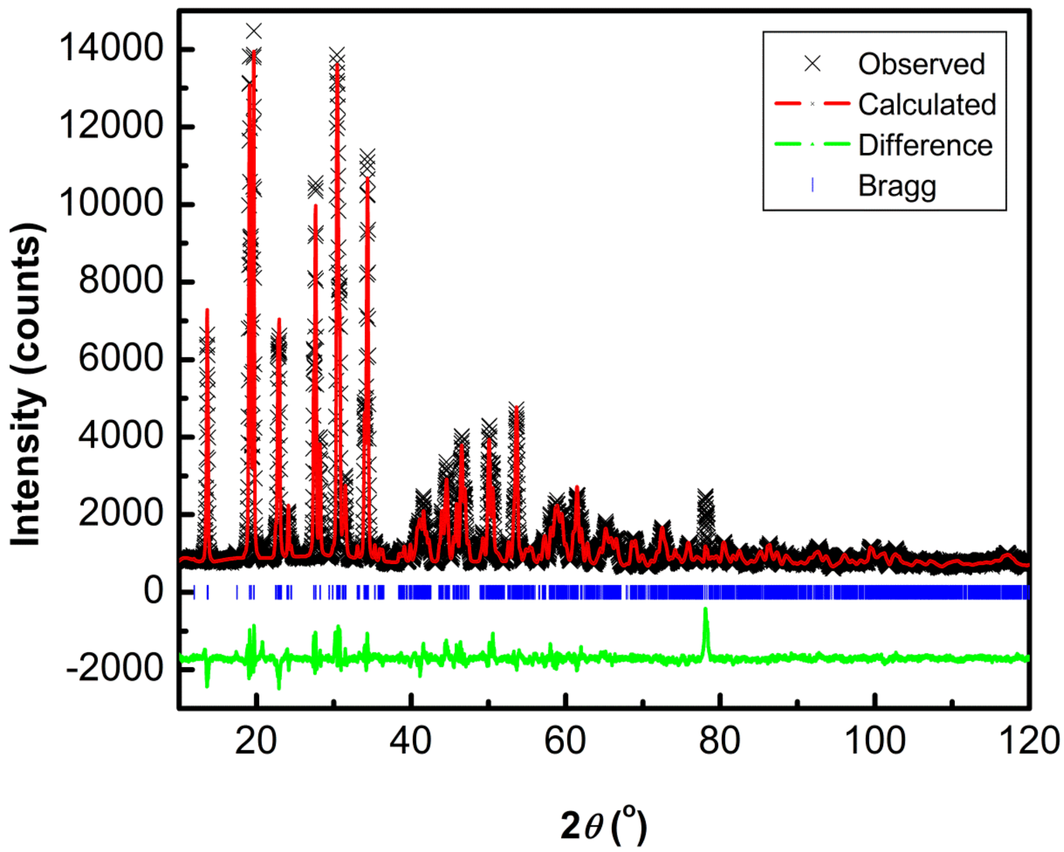


**Figure S1. Rietveld refinement plots for the result of $Na_{3.1}Zr_{1.95}Mg_{0.05}Si_2PO_{12}$ from powder XRD data.** The observed pattern, calculated profile, difference curve and the ticks for the Bragg reflections are plotted.

**Table S1. Results of the refinements of the structural parameters for the $Na_{3.1}Zr_{1.95}Mg_{0.05}Si_2PO_{12}$.**

| Atom | Occupation | x | y | z | $U_{iso}$ |
|---|---|---|---|---|---|
| Zr | 0.975 | 0.1011(83) | 0.2465(36) | 0.0551(3) | 0.023(55) |
| Mg | 0.025 | 0.7174(48) | -0.6058(96) | 1.9590(89) | 0.09 |
| P1 | 0.5 | 0 | 0.0437(66) | 0.25 | 0.107(61) |
| P2 | 0.25 | 0.3645(53) | 0.0744(24) | 0.2539(94) | 0.003(65) |
| Si1 | 0.56 | 0 | 0.0366(75) | 0.25 | 0.001(38) |
| Si2 | 0.72 | 0.3550(82) | 0.1134(42) | 0.2608(09) | 0.015(05) |
| Na1 | 0.230(7) | 0.25 | 0.25 | 0.5 | 0.077(64) |
| Na2 | 1.0 | 0.5 | 0.8867(02) | 0.25 | 0.019(89) |
| Na3 | 0.934(6) | 0.8172(9) | 0.1118(46) | 0.8763(76) | 0.205(44) |
| O1 | 1.0 | 0.1438(26) | 0.4457(79) | 0.2201(23) | 0.071(28) |
| O2 | 1.0 | 0.4365(77) | 0.4412(99) | 0.0839(59) | 0.037(81) |
| O3 | 1.0 | 0.2603(09) | 0.1786(29) | 0.2232(71) | 0.035(13) |
| O4 | 1.0 | 0.3732(54) | 0.1329(17) | 0.0954(66) | 0.030(66) |
| O5 | 1.0 | 0.4418(45) | 0.1767(71) | 0.4268(87) | 0.030(02) |
| O6 | 1.0 | 0.0888(22) | 0.1476(99) | 0.2506(79) | 0.010(25) |
| Agreement factors: $R_p$=5.13%, $R_{wp}$=7.39%, $Chi^2$=6.749 | | | | | |
| $a$=15.6600(96) Å, $b$=9.0613(93) Å, $c$=9.2272(28) Å, $V$=1089.17(6) $Å^3$ | | | | | |
| Phase weight fractions for phase no. 1 (NASICON): 0.946(25)<br>Phase weight fractions for phase no. 2 ($ZrO_2$): 0.0537(54) | | | | | |

**Table S2. Results of the refinements of the structural parameters for the $Na_{3.1}Zr_{1.95}Ca_{0.05}Si_2PO_{12}$.**

| Atom | Occupation | x | y | z | $U_{iso}$ |
|---|---|---|---|---|---|
| Zr | 0.975 | 0.1011(11) | 0.2483(44) | 0.0556(92) | 0.025(58) |
| Ca | 0.025 | 0.1785(67) | 0.1051(71) | -0.2421(78) | 0.075(04) |
| P1 | 0.5 | 0 | 0.0486(63) | 0.25 | 0.184(45) |
| P2 | 0.25 | 0.3649(07) | 0.0773(62) | 0.2618(29) | 0.033(82) |
| Si1 | 0.476(1) | 0 | 0.0268(71) | 0.25 | 0.007(58) |
| Si2 | 0.762 | 0.3556(53) | 0.1113(54) | 0.2610(97) | 0.020(98) |
| Na1 | 0.230(7) | 0.25 | 0.25 | 0.5 | 0.127(78) |
| Na2 | 1.0 | 0.5 | 0.8927(49) | 0.25 | 0.016(95) |
| Na3 | 0.934(6) | 0.8158(58) | 0.1184(75) | 0.9049 (61) | 0.254(02) |
| O1 | 1.0 | 0.1449(86) | 0.4325(38) | 0.2074(99) | 0.113(62) |
| O2 | 1.0 | 0.4399(25) | 0.4413(58) | 0.0744(92) | 0.026(27) |
| O3 | 1.0 | 0.2542(04) | 0.1797(75) | 0.2034(33) | 0.012(67) |
| O4 | 1.0 | 0.3756(15) | 0.1361(75) | 0.0911(97) | 0.043(39) |
| O5 | 1.0 | 0.4417(21) | 0.1832(91) | 0.4325(95) | 0.050(25) |
| O6 | 1.0 | 0.0865(52) | 0.1355(38) | 0.2485(83) | 0.013(49) |
| Agreement factors: $R_p$=5.21%, $R_{wp}$=6.92%, $Chi^2$=5.770 | | | | | |
| $a$=15.6671(42) Å, $b$=9.0634(97) Å, $c$=9.2282(29) Å, $V$=1089.77(1) $Å^3$ | | | | | |
| Phase weight fractions for phase no. 1 (NASICON): 0.947(02)<br>Phase weight fractions for phase no. 2 ($ZrO_2$): 0.0529(79) | | | | | |

**Table S3. Results of the refinements of the structural parameters for the $Na_{3.1}Zr_{1.95}Sr_{0.05}Si_2PO_{12}$.**

| Atom | Occupation | x | y | z | $U_{iso}$ |
|---|---|---|---|---|---|
| Zr | 0.975 | 0.1010(18) | 0.2462(42) | 0.0560(05) | 0.019(33) |
| Sr | 0.025 | 0.1853(31) | 0.0471(84) | -0.2503(61) | 0.029(99) |
| P1 | 0.5 | 0 | 0.0450(11) | 0.25 | 0.061(71) |
| P2 | 0.25 | 0.3645(99) | 0.0859(99) | 0.2576(64) | 0.004 |
| Si1 | 0.476(1) | 0 | 0.0345(65) | 0.25 | 0.022(19) |
| Si2 | 0.762 | 0.3566(59) | 0.1122(72) | 0.2608(59) | 0.019(24) |
| Na1 | 0.230(7) | 0.25 | 0.25 | 0.5 | 0.081(65) |
| Na2 | 1.0 | 0.5 | 0.8878(06) | 0.25 | 0.020(03) |
| Na3 | 0.934(6) | 0.8193(79) | 0.1201(13) | 0.9050(26) | 0.229(74) |
| O1 | 1.0 | 0.1406(03) | 0.4384(4) | 0.2145(65) | 0.053(4) |
| O2 | 1.0 | 0.4402(27) | 0.4435(04) | 0.0798(59) | 0.021(59) |
| O3 | 1.0 | 0.3716(62) | 0.1304(79) | 0.0943(25) | 0.047(88) |
| O4 | 1.0 | 0.3732(54) | 0.1329(17) | 0.0954(66) | 0.022(45) |
| O5 | 1.0 | 0.4473(42) | 0.1868(05) | 0.4363(9) | 0.036(04) |
| O6 | 1.0 | 0.0840(16) | 0.1481(2) | 0.2455(87) | 0.001(12) |
| Agreement factors: $R_p$=5.06%, $R_{wp}$=7.04%, $Chi^2$=5.942 | | | | | |
| $a$=15.6623(36) Å, $b$=9.0628(78) Å, $c$=9.2289(32) Å, $V$=1089.70(4) Å$^3$ | | | | | |
| Phase weight fractions for phase no. 1 (NASICON): 0.968(37)<br>Phase weight fractions for phase no. 2 ($ZrO_2$): 0.0316(29) | | | | | |

**Table S4. Results of the refinements of the structural parameters for the $Na_{3.1}Zr_{1.95}Ba_{0.05}Si_2PO_{12}$.**

| Atom | Occupation | x | y | z | $U_{iso}$ |
|---|---|---|---|---|---|
| Zr | 0.975 | 0.1012(35) | 0.2459(6) | 0.0557(68) | 0.021(21) |
| Ba | 0.025 | 0.1634(73) | 0.0826(31) | -0.3217(23) | 0.059 |
| P1 | 0.5 | 0 | 0.0500(71) | 0.25 | 0.030(44) |
| P2 | 0.25 | 0.3585(47) | 0.0961(38) | 0.2602(68) | 0.002(06) |
| Si1 | 0.56 | 0 | 0.0388(15) | 0.25 | 0.045 |
| Si2 | 0.72 | 0.3569(76) | 0.1135(97) | 0.2600(21) | 0.020(58) |
| Na1 | 0.230(7) | 0.25 | 0.25 | 0.5 | 0.104(86) |
| Na2 | 1.0 | 0.5 | 0.8869(12) | 0.25 | 0.021(41) |
| Na3 | 0.934(6) | 0.8211(62) | 0.1186(17) | 0.9124(48) | 0.220(02) |
| O1 | 1.0 | 0.1384(08) | 0.4416(45) | 0.2108(82) | 0.053(12) |
| O2 | 1.0 | 0.4425 | 0.4418(19) | 0.0820(13) | 0.024(17) |
| O3 | 1.0 | 0.2572(79) | 0.1814(45) | 0.2141(64) | 0.042(32) |
| O4 | 1.0 | 0.3689(86) | 0.1314(08) | 0.0936(75) | 0.018(35) |
| O5 | 1.0 | 0.4466(76) | 0.1880(77) | 0.4347(48) | 0.028(12) |
| O6 | 1.0 | 0.0848(32) | 0.1498(73) | 0.2461(17) | 0.001(38) |
| Agreement factors: $R_p$=5.26%, $R_{wp}$=7.34%, $Chi^2$=6.453 | | | | | |
| $a$=15.6543(43) Å, $b$=9.0581(51) Å, $c$=9.2242(98) Å, $V$=1088.03(6) $Å^3$ | | | | | |
| Phase weight fractions for phase no. 1 (NASICON): 0.967(55)<br>Phase weight fractions for phase no. 2 ($ZrO_2$): 0.0324(49) | | | | | |
